# Care during Breastfeeding: Perceptions of Mothers and Health Professionals

**DOI:** 10.17533/udea.iee.v37n2e09

**Published:** 2019-09-19

**Authors:** Camila Lucchini-Raies, Francisca Márquez-Doren, Nicole Garay Unjidos, Javiera Contreras Véliz, Daniel Jara Suazo, Cristina Calabacero Florechaes, Solange Campos Romero, Olga Lopez-Dicastillo

**Affiliations:** 1 Nurse Midwife, MNSc., Ph.D. candidate. Associate Professor, Pontificia Universidad Católica de Chile Email: clucchin@uc.cl Pontificia Universidad Católica de Chile Pontificia Universidad Católica de Chile Chile clucchin@uc.cl; 2 Nurse Midwife. MNSc., Ph.D. Associate Professor, Pontificia Universidad Católica de Chile. Email: fmarquez@uc.cl. Corresponding author. Pontificia Universidad Católica de Chile Pontificia Universidad Católica de Chile. Chile fmarquez@uc.cl.; 3 Nurse, MSP. Assistant Professor, Pontificia Universidad Católica de ChileEmail: nggaray@uc.cl Pontificia Universidad Católica de Chile Pontificia Universidad Católica de Chile Chile nggaray@uc.cl; 4 Nurse. Pontificia Universidad Católica de Chile. Email: jpcontr3@uc.cl Pontificia Universidad Católica de Chile Pontificia Universidad Católica de Chile Chile jpcontr3@uc.cl; 5 Nurse Midwife. Adjunct Instructor, Pontificia Universidad Católica de Chile. E-mail: dsjara@uc.cl Pontificia Universidad Católica de Chile Pontificia Universidad Católica de Chile Chile dsjara@uc.cl; 6 Nurse. Pontificia Universidad Católica de Chile. Email: ccalabacero@med.puc.cl Pontificia Universidad Católica de Chile Pontificia Universidad Católica de Chile Chile ccalabacero@med.puc.cl; 7 Nurse Midwife. MSP., Ph.D. Associate Professor, Pontificia Universidad Católica de Chile. E-mail: scamposr@uc.cl Pontificia Universidad Católica de Chile Pontificia Universidad Católica de Chile Chile scamposr@uc.cl; 8 Nurse, MSc. Ph.D. Professor, Universidad de Navarra, Spain. Email: olopezde@unav.es Universidad de Navarra Universidad de Navarra Spain olopezde@unav.es

**Keywords:** breastfeeding, mothers, primary care nursing, qualitative research., aleitamento materno, mães, enfermagem de atenção primária, pesquisa qualitativa.

## Abstract

**Purpose.:**

To know the perceptions of mothers and health professionals in relation to the care provided and received during breastfeeding at primary health care level.

**Methods.:**

A qualitative exploratory study was conducted with breastfeeding mothers (10) and primary health care professionals (24). Data was gathered through in-depth interviews and focus groups. Data analysis was performed through thematic content analysis. The rigor of the study was ensured by the Guba and Lincoln criteria for qualitative research. Ethical aspects were addressed through the informed consent process, confidentiality, and methodological rigor.

**Results.:**

The experience of providing/receiving breastfeeding support was revealed as a dynamic, multidimensional care and support process, through three central themes: 1. Influence of previous care and support experiences during the breastfeeding process; 2. Importance of the context within which care is framed; and 3. Addressing emotions to establish trust between professionals and mothers.

**Conclusion.:**

The study findings contribute to further understanding a complex phenomenon, such as breastfeeding support and care for mothers/families, from the experience of the actors involved, deepening the experiences of both in integrated manner. In addition, the relational, organizational, and contextual dimensions that influence support, and that should guide care, are also highlighted.

## Resumo

**Objetivo.:**

Conhecer as percepções de mães e profissionais de saúde em relação aos cuidados que se brindam e recebem durante o processo de amamentação no nível primário de atenção.

**Métodos.:**

Estudo qualitativo exploratório realizado em Santiago (Chile) com a participação de 10 mães em processo de amamentação e 24 profissionais da saúde. O recolhimento de dados se realizou através de entrevistas em profundidade e grupos focais. A análise da informação se obteve através da análise de conteúdo temático. O rigor da investigação se cautelou por meio dos critérios de Guba e Lincoln. Os aspectos éticos se abordaram mediante o processo de consentimento informado, confidencialidade e rigor metodológico.

**Resultados.:**

A experiência de brindar e receber apoio durante o processo de amamentação se revelou como um processo de cuidado e apoio dinâmico, multidimensional, através de três temas centrais: Influência das experiências prévias de cuidado e apoio durante o processo de amamentação; Importância do contexto no qual se enquadra o cuidado; e Abordagem das emoções para o estabelecimento da confiança entre profissionais e mães.

**Conclusão.:**

As percepções de mães e profissionais de saúde em relação aos cuidados que se brindam e recebem durante o processo de amamentação são um fenômeno com dimensões contextuais, organizacionais e relacionais que influem no apoio e que deveriam orientar o cuidado.

## Introduction

Breastfeeding is considered the optimal form of infant feeding exclusively up to six months of life and then complemented, up to two years of age or more.(1) In Chile, exclusive breastfeeding at six months of life is at 53%,(2) a figure that has increased due to strategies implemented for its promotion. Although current figures are close to the expected goal (60%), the percentage of children with exclusive breastfeeding at one month of life is the lowest since 1993.(3) Nationally, policies and programs exist focused on favoring the biopsychosocial development of children, which contemplate promotion of breastfeeding.(4) Evidence exists about the sociodemographic characteristics of breastfeeding women; rates of initiation of breastfeeding, its duration, main causes for weaning, and the most-frequent problems, among others.(5,6) Likewise, some aspects of the social environment, like the support received by the mothers from the health staff,(7) and the self-efficacy of women in relation to breastfeeding.(8) Thus, it is known that factors influencing upon the breastfeeding process are known partially, which hinders its establishment and maintenance, and the design of strategies by the health staff.(9) 

With respect to how health professionals experience the process of providing support to mothers who breastfeed, an inconsistency exists, characterized by contradictory accounts in relation to breastfeeding, which creates confusion in the mothers. The aforementioned is related with the support to breastfeeding being a dynamic, multidimensional process with diverse relational, contextual, and situational components.(10) The purpose of this research was to know the perceptions of mothers and health professionals in relation to the care provided and received during the breastfeeding process in the primary care level. 

## Methods

A qualitative exploratory study was conducted in two Family Health Centers of primary care level in the Metropolitan Region of Santiago, Chile. Through a purposeful sampling, 45 health professionals and 20 mothers were invited to participate in the study of which 24 professionals (20 who provided care to mothers in the breastfeeding process and four who worked in management positions), and 10 breastfeeding women accepted participation in the study. The inclusion criteria of the professionals were: provide direct care to the mother/child during the gestation, puerperium, and infant health supervision; and professionals in management positions related with breastfeeding decision making. The inclusion criteria for the mothers were: 18 years and older, breastfeeding her child under 1 year of age, primiparous and multiparous. Maternal contraindication to breastfeeding and pre-term children or with pathologies that interfere with establishing the breastfeeding practice were considered exclusion criteria. 

The principal researcher invited, through e-mail, professionals who provided care to participate in a focus group, and the managers to an in-depth interview. The mothers were invited to an in-depth interview by a nurse from each Health Center. Prior to the activities to collect information, the research team explained to the participants the objectives and ethical considerations of the study and, thereafter, the participants signed the informed consent. 

The number of participants was determined through data saturation, which was reached with two focus groups of direct care professionals, four in-depth interviews with the managers, and 10 in-depth interviews with the mothers, for a total of 34 participants. The interviews and focus groups were conducted during October 2017 and January 2018 by the principal researcher and a co-researcher, who kept a record through a field diary of their own feelings and experiences on the study theme. To collect the sociodemographic characteristics of the mothers and the professionals, questionnaires were used created specifically for such. Interviews with the mothers were conducted in the Health Centers, in a place specifically assigned for said purpose, with an average duration of 30 minutes, starting with the question: What has been your experience with breastfeeding your child from the beginning until now? 

For the professionals who provided direct care, a focus group was conducted in each Health Center, which had a script of questions established and lasted 60 minutes each. Finally, in-depth interviews were carried out with the management professionals, starting with the question: What does the organization currently do to support mothers and their families during the breastfeeding process? The interviews and the focus groups were audio-recorded, transcribed textually, and anonymized for subsequent coded storage in the principal researcher’s computer. 

The Scientific Ethics Committee of the Faculty of Medicine at Pontificia Universidad Católica de Chile and the Ethics Committee of the South East Metropolitan Health Service of Santiago de Chile approved the study. A thematic content analysis was performed, using Dedoose software, generating a detailed and systematic record of the themes and common aspects of the reports by the mothers and the professionals, grouping such into higher-order categories and subcategories.(11) Compliance of the criteria of methodological rigor was ensured for qualitative research proposed by Guba and Lincoln.(12) 

To present the results, the following abreviations were used to identify the source of information; when interviewing the mothers, the abreviation MI was used followed by the number of the interview (for example: MI7 is the seventh interview carried out with mothers). For interviews conducted with the professionals in management positions, the abbreviation MPI was used. Likewise, for the focus groups with the professionals, the abreviation used was FG, followed by abreviations accounting for the type of professional cited: Midwife (M), Nurse (N), and Family Physician (FP). Thus, for example, FGM means that said citation was obtained during the focus group and corresponds to the Midwife professional.

## Results

The principal characteristics of the 24 health professionals and the 10 mothers participating in the study can be observed in Table 1.

**Table 1:** Principal characteristics of the study participants

Characteristic	Value
Health staff (n=24)	
Gender	
Female	21/24
Male	3/24
Age range in years	25 to 43
Profession	
Physician	12/24
Nurse	8/24
Midwife	4/24
Level of formation	
Undergraduate	9/24
Graduate	15/24
Professional experience in years	1-19
Personal experience of motherhood/fatherhood	
Yes	11/24
No	13/24
Personal experience or of their partner with breastfeeding	11/24
Perception with experience of breastfeeding	
Very good/good	9/11
Poor	2/11
Mothers (n=10)	
Age range in years	21-32
Education > 10 years	8/10
Presence of the partner	10/10
Prior experience with breastfeeding	
Yes	6/10
Duration in months	8-36
It was a good experience	6/6
Reasons for weaning	
Return to work	3/6
Spontaneous by the child	2/6
Difficulty sleeping	1/6
Type of delivery	
Vaginal	5/10
Cesarean	5/10
Early skin-to-skin contact	5/10
Begin breastfeeding first hour of life	2/10
Mother and child rooming-in	8/10
Supplementation with formula during puerperium due to difficulty in latching	7/10
Exclusive breastfeeding upon discharge	8/10
Child’s type of feeding at the moment of the interview	
Less than 6 months with exclusive breastfeeding	4/6
Less than 6 months without exclusive breastfeeding	2/6
Over 6 months with breastfeeding + solid feeding	3/4
Over 6 months with mixed breastfeeding +solid feeding	1/4

The experience of providing and receiving support during the breastfeeding process was revealed as a process of care and dynamic and multidimensional support. This experience was classified into three central themes: *the influence of previous care and support experiences during the breastfeeding process; importance of the context in which care is framed; and addressing emotions to establish trust between professionals and mothers.*

### Influence of previous care and support experiences during the breastfeeding process

The actors involved in the process show up with their host of experiences in relation to breastfeeding, from a personal and professional dimension, where their own experiences are conjugated with breastfeeding care and support experiences during this process. Satisfaction or difficulty felt by the mothers with respect to breastfeeding at that very moment is the starting point: *The truth is she accepted the breast immediately, the midwife told me: “it’s as if she had always been breastfed”, it did not take anything… I felt no difficulty (MI3)*. The mothers express specific support demands, related with a need of being cared for to improve their own care of their children and the breastfeeding process. The needs emerging from the diverse realities the women experience require family support, with a leading role gained by the partner and the mother, who are constituted into their principal figures of support. The experiences of the professionals who determine how support is being provided to mothers/families in the breastfeeding process emerge from the experiences in relation to their own breastfeeding process, and from the experiences around care and previous support offered within the health care context: *My own breastfeeding experience I believe is super important when supporting other mothers because I also had some difficulties… to be able to empathize with another (FGFP)*. 

Furthermore, professionals take into consideration their prior care and support experiences with breastfeeding, which nourish their way of confronting new instances of support: *There is an important link here with the patients, generally when they are multiparous we work with the way it went in previous pregnancies, how did it go with the breastfeeding… to prepare us for whatever is coming and to break the myth that if it was bad in previous pregnancies, now it will not be necessary for the same to occur (FGM).*

### Importance of the context in which care is framed

Those contextual situations that intervene in the care experience related with the institution and professionals, take place within a scenario of primary level health care. Professionals, as well as mothers/families, have the possibility of interacting in diverse instances of the breastfeeding process, propitiating the interaction and links among them. In turn, professionals highlight tools at care management level, like the existence of programs and policies that promote support for breastfeeding at country level; and orientations that guide the management of the organization of care: *I think that from the organizational point of view and - above all - from the perspective of the infant program, which we are in charge of promoting breastfeeding, is to be constantly training and reminding, enhancing the importance of breastfeeding (MPI1).* Additionally, professionals emphasize elements to support breastmilk, such as the contents and modality addressed, as aspects that are part of their context and which could favor breastfeeding. Themes are highlighted, like the technique and positions of breastfeeding, demystification of beliefs, technique of breastmilk extraction, benefits of breastfeeding, among others: *What we see are the benefits of breastfeeding; we speak ideally of the minimum ages it is recommended… specifically the latching, the positions that can be tried. Ah, and we see extraction of breastmilk, duration times, how to store it (FGN).*

Also, as part of the institutional context, the participants identify the existence of an institutional conviction about the relevance of *breastfeeding* during the development of infant health and the importance of promoting it through programmed support activities: *There is great disposition for teamwork and to promote these support interventions. Everyone has it quite clear that with breastfeeding there is lower risk of acute infections, less risk of obesity (MI2)*. Likewise, mothers also identify the emphasis of breastfeeding by the health staff as a strength: *In reality, I have always seen here a special emphasis on breastfeeding. Support is very good from everyone (MI3).* In relation to the contextual situations of the families that impact upon the support, the professionals indicate that these are related with the psychosocial conditions of the population in which the health centers are inserted. For example, that mothers with informal jobs, without postnatal rest, are obligated to return to labor activities early, or the consumption of drugs by the mother, as situations that impede prolonging the breastfeeding through the time recommended: *Because we have many mothers who are not with their children after two months because they need to return to work… This also happens with drug consumption, within a highly vulnerable social setting that hinders maintaining the breastfeeding activity for the greatest time possible (MPI2).*

### Addressing emotions to establish trust between professionals and mothers

Care and support activities during the breastfeeding process are experienced through an interpersonal professional-mother/family encounter, whose key elements are the recognition of the emotions involved and the establishment of trust between the actors. The mothers indicate experiencing initially negative emotions in relation to breastfeeding, such as affliction for not being able to breastfeed, physical discomfort with breastfeeding, frustration for feeling that they are not doing it well, and fear of breastfeeding: *I suffered it a lot because sometimes I felt I could not feed, I got desperate and I would start to cry. I was desperate because I would say: “oh heck, I can’t breastfeed my child because my nipples are very short, they are small” (MI7)*. These feelings are more present at the beginning of breastfeeding and disappear as this process progresses and the mother overcomes the difficulties. The mothers also identify feelings of happiness and satisfaction for having achieved to breastfeed, which remains as global feeling of the experience, even when initially the emotions tend to be more negative. The professionals, in turn, perceive that mothers go through a process that is variable and particular, but which in general most of them are willing to breastfeed, given that they know that it is best for their children. Nevertheless, they perceive that the first weeks postpartum constitute a period during which mothers experience anguish and lack of support; and then they become empowered until they manage to enjoy it: *But I believe that for all of them it is difficult, above all with the first or second baby, it is always difficult (FGM).*

From the recognition of the emotions involved, it is possible to establish the encounter and identify the needs of the mothers/families for which it becomes necessary to promote a safe and trusting environment. The mothers are capable of identifying in the professionals those positive and negative characteristics that impact significantly on their perception of support and on how the encounter and the trust between them is generated. As elements that strengthen support, they emphasize the close, respectful, and affectionate treatment they perceived from the professionals; their disposition to clear doubts, consider their opinion and experience, and provide information. These attributes favor actors strengthening mutual trust during the interpersonal encounter: *I think it is also in how you are given the information; here, they are quite close, they are respectful. We were surprised, for example, that when they call a patient, they greet them with a kiss, “hi, how are you?” or they shake hands, very personalized (MI6).* In addition, the mothers were also capable of identifying those characteristics from the professionals who did not provide them support, and who had a negative influence on their experience. They indicate that the members of the health staff who did not support them were characterized by a distant and cold treatment, with little or no disposition to clear their doubts, with lack of time to listen to them, and who pressured them to breastfeed without considering what was happening to them: *Because there are some doctors or midwives who are very grim, very cold and who do their work and bye (MI7).* To the extent to which a relationship was established based on security and trust, the mother perceived that the professional was close and was focused on supporting her to satisfy her own needs. The aforementioned seemed to favor increased trust in relation to their capacity to breastfeed. Likewise, to the extent to which the professional perceived being in tune with the mother with respect to the care offered, trust also seemed to increase in relation to their capacity to support the mother/family during the breastfeeding process. On the contrary, when the professional-mother/family relationship was established, on an environment of distrust and insecurity, the mother perceived that the professional was not focused on supporting her to satisfy her needs, causing more anguish and distancing. In turn, the professionals perceived difficulty to begin the relationship and doubted of their capacities to support.

## Discussion

The interpersonal relationship between health professionals and mothers/families is the starting point to establish care interactions. Nurses conceive these relationships as the means to provide care centered on the unique needs of each person.(13) This is how, through the interpersonal relationship, the complexity of nursing care is expressed, considering the dimensions in its human and social nature, as in this study. An aspect that characterizes the relationship established between the professional and the mother/family in breastfeeding support is that it is strongly influenced by the personal and professional experience of the actors involved, which has also been reported by other authors.(14) This could be explained because breastfeeding is a social process developed in diverse individual and collective settings and which is much more than the promotion of a health behavior. 

The results of this study show that the relationship between the professional and the mother/family during the breastfeeding process is characterized by being an encounter of multidimensional care where the professional supports the mother/family for them - in turn - to care for the child, conceiving this encounter as a constellation of care. Although some studies highlight the importance for the mother to receive support from professionals and their family,(6,15) no publications were found that conceptualize the integration of the different types of support, addressing this constellation of care produced. Previous studies have identified that mothers go through a cascade of negative feelings that evolve toward positive feelings as the mother gains trust in the breastfeeding process.(16) During this process, the mother constitutes her maternal identity and, hence, the support she receives from professionals becomes relevant.(10) This study observed how mothers have felt judged by the health staff when having difficulties in breastfeeding their children; others, on the contrary, felt the support received allowed them to gain trust in their breastfeeding process.

The finding that the personal experience of health professionals with their own breastfeeding influence on the care they provide is of great interest. Within the breastfeeding setting, it has been described that professionals after having lived their own experiences, changed their way of providing care.(17) These findings do not seem exclusive of this type of care; rather, they extend to other health promotion environments.(18) This seems to indicate that addressing the personal experiences of professionals with respect to care in health promotion, and specifically in the setting of breastfeeding, could be essential when proposing improvements in the care they provide. In addition, this study found that professional experience in care for breastfeeding impacts upon the support offered to the mothers. The participants reported having had prior positive care experiences that reinforced positively their attitudes and beliefs regarding the support they provided to the mothers. Similarly, other studies endorse this idea, but in negative sense. When professionals have experienced difficulties in providing care to breastfeeding mothers, they have identified lack of preparation(19) or have even concluded that it is not part of their professional role to offer this type of support.(20) This could be distancing them from providing the care the mothers need. 

The mothers and professionals in this study identified conditions arising during the encounter that permitted them to give meaning to the experience of care and support. Among those conditions that permit giving a positive meaning to the experience of the encounter, trust in the professional-mother/family relationship emerges as a relevant aspect. The aforementioned is backed by studies that indicate that establishing trust in the relationships between nurses and patients promotes commitment and improves the disposition of patients to be active members within the care team.(21) Conversely, when a climate of trust is not established, the encounter can be weakened and transformed into a negative experience for both actors. Some of the conditions identified by the participants in this study that contribute to distrust in the interpersonal relationship are lack of respect for the decisions made or for the situation each mother is in, not including the partner, and lack of professional skills aimed to effective communication in the professional-patients relationship. 

Other studies have described some essential attributes for trust to exist in the nurse-patients relationship that could be applied in the case of breastfeeding. Among them, we can highlight the ability of professionals to establish relationships through effective communication, which will allow them to identify the unique needs of each person.(21) Thus, this study has observed that to the extent that a relationship was established based on trust, the mother perceived the professional as another close person. Thus, mothers seemed to increase trust in relation to their capacity to breastfeed. Reciprocally, when professionals perceived being in tune in the relationship with the mother, they also seemed to increase their trust in relation to their capacity to support them during their breastfeeding process. This finding is related directly with the concept of self-efficacy, which corresponds to the perception people have about their own capacities to achieve certain performances.(22) Self-efficacy has been applied to the breastfeeding process and evidence shows that the greater the trust the mother has in her capacity to breastfeed, greater will be the possibility for successful breastfeeding. It may be due to this that interventions that include this concept to the support mothers during the breastfeeding process have proven effective in improving rates of exclusive breastfeeding.(23) Further, in relation to the self-efficacy perceived by the professionals, this seems to increase as they acquire more experience in support and also when they themselves have experienced the process of breastfeeding a child.(24) Both aspects are of interest for the proposal of opportunities in training professionals to improve exposure to significant learning experiences that permit them to increase their self-efficacy without having to depend on their personal experiences with breastfeeding. 

This study also identified the importance of the context in which the professional-patient encounter takes place. Existence of activities to support multilevel breastfeeding backed and framed within specific policies increases the impact on exclusive breastfeeding rates and on its duration.(25) Although in recent decades many efforts have been invested to better understand the effectiveness of different support interventions for breastfeeding;(5,25) few studies have focused on understanding relational, organizational, and contextual aspects that could impact upon this practice and, thereby, on the care required to support it.(26) 

The results herein contribute to understanding how the context could influence positively or negatively on the results of interventions implemented to support breastfeeding. In this study, some of the participating mothers belonged to groups with social vulnerability and/or from diverse origins, which supposes an added challenge for health professionals. To individualize care from a sociocultural perspective, professionals need to establish a facilitating relationship, show respect for the beliefs and values of the women and their family, and support making informed decisions by them. (27) 

In conclusion, knowledge of the perceptions of mothers and health professionals in relation to care provided and received during the breastfeeding process was revealed as a critical dimension that must be considered to provide care centered on the needs of those who receive it and those who provide it. The results reported by this study contribute to delve into understanding a complex phenomenon, like support and care of mothers/families during the breastfeeding process, from the experiences of the actors involved, with an integral perspective, given that these delve into the experiences of both in interrelated manner. Furthermore, the study reveals the contextual, organizational, and relational dimensions that influence upon the support and which should guide care. 

The study presented limitations worth mentioning. One was that it did not consider participation from the closest relatives, who turned out to be the principal sources of support for the mothers. The work included participating mothers who belonged to a lower-middle and middle class, who were users of the public health system (majority of the Chilean population). However, there is a percentage of the population of which little is known, which has acces to health care in the private sector and for the same reason, does not have direct access to public policies in support of breastfeeding. Given the aforementioned, we suggest continuing with additional research that includes significant participants for the mothers and who belong to both health systems (public and private), to delve into the experience of care in relation to breastfeeding and learn from their own perspective how this phenomenon is experienced in a broader manner. 
